# Continuous cuffless blood pressure monitoring with a wearable ring bioimpedance device

**DOI:** 10.1038/s41746-023-00796-w

**Published:** 2023-03-30

**Authors:** Kaan Sel, Deen Osman, Noah Huerta, Arabella Edgar, Roderic I. Pettigrew, Roozbeh Jafari

**Affiliations:** 1 Department of Electrical and Computer Engineering, Texas A&M University, College Station, TX USA; 2 Department of Mechanical Engineering, Texas A&M University, College Station, TX USA; 3 Department of Biomedical Engineering, Texas A&M University, College Station, TX USA; 4 School of Engineering Medicine, Texas A&M University, Houston, TX USA; 5 Department of Computer Science and Engineering, Texas A&M University, College Station, TX USA

**Keywords:** Diagnostic markers, Hypertension, Electrical and electronic engineering, Translational research

## Abstract

Smart rings provide unique opportunities for continuous physiological measurement. They are easy to wear, provide little burden in comparison to other smart wearables, are suitable for nocturnal settings, and can be sized to provide ideal contact between the sensors and the skin at all times. Continuous measuring of blood pressure (BP) provides essential diagnostic and prognostic value for cardiovascular health management. However, conventional ambulatory BP measurement devices operate using an inflating cuff that is bulky, intrusive, and impractical for frequent or continuous measurements. We introduce ring-shaped bioimpedance sensors leveraging the deep tissue sensing ability of bioimpedance while introducing no sensitivity to skin tones, unlike optical modalities. We integrate unique human finger finite element model with exhaustive experimental data from participants and derive optimum design parameters for electrode placement and sizes that yields highest sensitivity to arterial volumetric changes, with no discrimination against varying skin tones. BP is constructed using machine learning algorithms. The ring sensors are used to estimate arterial BP showing peak correlations of 0.81, and low error (systolic BP: 0.11 ± 5.27 mmHg, diastolic BP: 0.11 ± 3.87 mmHg) for >2000 data points and wide BP ranges (systolic: 89–213 mmHg and diastolic: 42–122 mmHg), highlighting the significant potential use of bioimpedance ring for accurate and continuous estimation of BP.

## Introduction

Smart rings with embedded smart sensors are a particularly prominent form-factor as they are easy to wear, not burdensome, and provide ideal sensor-to-skin contact, making them appropriate for seamless operation in outpatient settings. The unobtrusive nature of the rings is especially valuable for taking nocturnal hemodynamic measurements, as they allow for uninterrupted measurements of cardiovascular health status while mitigating interference from the external stimuli present during waking hours that may artificially alter the physiological state, such as stress and physical exertion^1^. Continuous monitoring of hemodynamic parameters plays a crucial role in improving diagnostic and prognostic medical care. Blood pressure (BP) is a key biomarker that is frequently used in non-ambulatory settings by medical professionals to assess cardiovascular status^2^. However, conventional ambulatory BP measurements are taken with a sphygmomanometer device operating on an inflating cuff that is bulky and intrusive. Therefore, long-term continuous BP measurements in daily, ambulatory, or nocturnal settings cannot be achieved with such systems^3,4^, due to the discomfort caused by inflating cuff as well as the bulkiness of the system^5,6^. Moreover, BP recordings taking place during clinic visits are exposed to a measurement bias (e.g., *white coat syndrome:* an artificial rise in BP in medical clinics, while normal at home, *masked hypertension:* BP recorded at normal ranges in clinics, while being high in ambulatory settings)^7^. Therefore, there is a need for non-invasive and unobtrusive technologies that can effectively capture blood flow and other important hemodynamic parameters to replace current traditional practice while introducing minimum discomfort for the users.

Certain wearable modalities, such as optical photoplethysmography (PPG) and bioimpedance-based wrist-worn systems, provide non-invasive and seamless operation. The PPG-based systems, however, suffer from signal quality due to the low penetration of light^8^ that is also highly sensitive to skin characteristics, e.g., varying skin-tones^9^, body-mass index^10^, skin-temperature^11^, and poor customizability. Bioimpedance-based systems overcome the limitations of optical systems as the modality depends on the distribution of a very minimal, non-invasive, high-frequency alternating current (AC) underneath the skin, and the recordings of the voltage signal based on the volumetric distribution of different tissue types with their unique electrical characteristics. Bioimpedance is shown to provide deep tissue sensing to effectively capture arterial blood flow when an array of bioimpedance electrodes builds a firm contact with the skin and is aligned with the underlying arteries^12^. Previous studies show application of bioimpedance at different body regions (e.g., chest^13^, wrist^14^, foot^15^) to capture physiological parameters such as heart rate (HR), respiration rate (RR), and leverage machine learning (ML) models to obtain beat-to-beat systolic (SBP) and diastolic blood pressure (DBP) values^16^. A major challenge with bioimpedance technique is to establish high contact quality through a reliable, long-lasting, and firm interface between the electrodes and the skin. This requires continuous attention from the users to the placement of sensors and creates additional challenges for the translation of this powerful technology to practice. Recent reports explore self-adhesive materials (e.g., 2D-layer graphene, ultra-thin gold electronic-tattoo) for bioelectronic measurements^17,18^. However, major issue with these materials is establishing a suitable connection between the flexible substrates and electronics which are rigid, and the connection often becomes the source of failure.

**Fig. 1:**
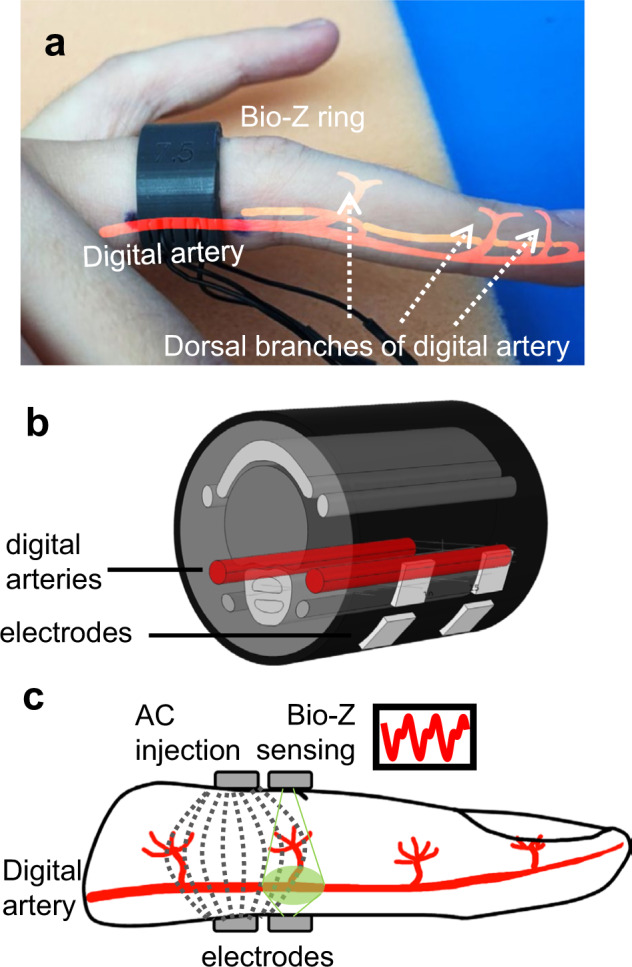
Bioimpedance (Bio-Z) ring sensor for arterial blood flow and pressure sensing. **a** A fabricated ring example custom fit to user’s finger size. **b** The finite element model of the human finger used in guiding ring design. **c** Cross-section of the four electrode bioimpedance sensing, with dashed lines representing high frequency alternating current (AC) distribution within the finger, and green area shows sensitive area for Bio-Z sensing electrodes.

In this paper, we propose a ring-shaped bioimpedance sensor that addresses the major challenge of establishing consistently high-quality contact with bioimpedance signal acquisition systems, as shown in Fig. 1. The design of rings is guided by a proof-of-concept study integrating bioimpedance model simulations from a unique finite element model of the human finger and thorough experimental analysis on human participants, including an analysis of continuous BP estimation. The ring sensors are fabricated with various dimensions to fit for the specific ring sizes of individuals participated in our experimental validation study. In addition to the guaranteed electrode-skin contact, the ring-sensors use a semi-flexible silicon material to further provide unobtrusive and seamless user experience to the wearers motivating long-term and nocturnal use among the users. The ring sensors include four small-sized (3 mm by 3 mm) silver electrodes to achieve non-invasive bioimpedance sensing. In addition, once research level investigation is finalized the rings can easily host the necessary hardware (a microcontroller—MCU chip with integrated analog-to-digital converter—ADC for bioimpedance signal sampling and digital-to-analog converter—DAC for programmable signal injection to skin, possibly a Bluetooth module for data transfer) and smart power management^19^ to provide a fully wearable bioimpedance operation.

The primary challenge with ring-based bioimpedance sensing is to achieve high sensitivity and specificity to the blood flow due to multiple reasons: (i) the digital arteries within the fingers receive their blood supply, that is still in the form of a pulsatile blood pressure wave^20^, from palmer arches connected to radial and ulnar arteries. The size of these digital arteries is much smaller than the rest of the arterial tree (typical diameter for common digital arteries is <2 mm^21^) allowing them to carry a fraction of blood volume changes per cardiac cycle, (ii) the available active space between electrodes and the skin within a ring-form factor is much less than within a wrist-form factor, therefore placement of all of the bioimpedance electrodes in a single line, along the artery for highest sensitivity is not feasible. We created our framework while remaining cognizant of these two major drawbacks with ring bioimpedance sensors: (i) we developed a finite element model (FEM) of finger considering human anatomy and unique electrical and dimensional characteristics of different tissue layers (i.e., skin, fat, tendon/ligament, nerve, artery). We conducted an electrical simulation on the model to assess the current distribution within the finger and the sensitivity obtained with the voltage-sensing electrodes at varying electrode placements, (ii) we designed a thorough set of experiments to validate our observations from the simulation platform with the participation of *N* = *5* healthy volunteers. We run statistical analysis on the beat-by-beat ensembled bioimpedance signals to quantify the sensor ability to capture blood pressure wave flow through the digital arteries, (iii) we built an ML model that estimates beat-to-beat BP readings with the data collected from *N* = *10* presumably healthy participants. During the data collection, we asked the participants to follow an exercise protocol to induce a change in their BP. This allows us to test the performance across wide ranges of pressure values (SBP: 89–213 mmHg and DBP: 42–122 mmHg) and achieve performance well within the range of the accuracy recommendations by the American Association for the Advancement of Medical Instrumentation (AAMI) for BP devices (mean error and standard deviation, SBP: 0.11 ± 5.27 mmHg, DBP: 0.11 ± 3.87 mmHg, see Supplementary Table 1)^22^.

## Results

### Finite element modeling and electrical current simulations of human ring finger

**Fig. 2:**
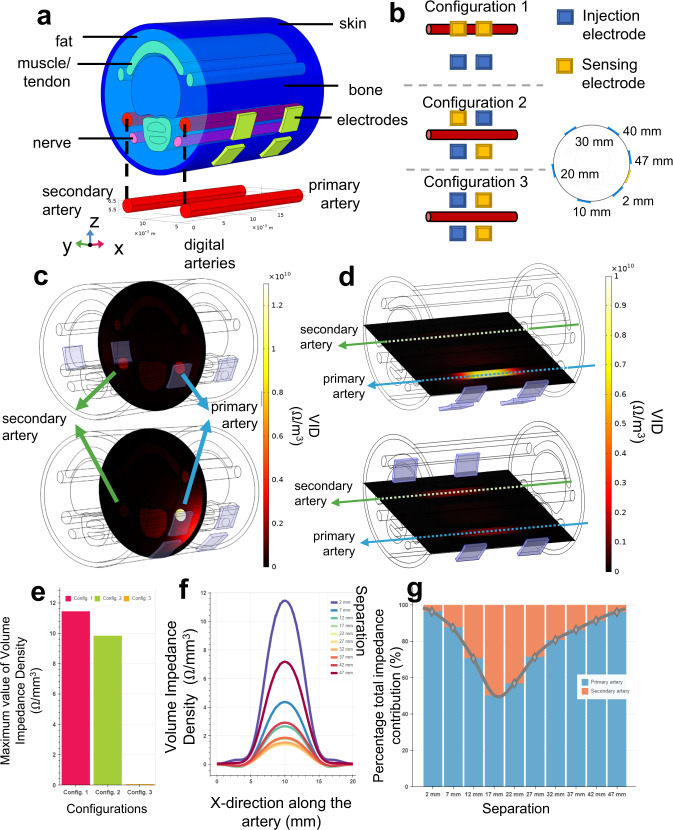
Finite element model of the finger and its electrical simulations. **a** Three-dimensional finger model scaled for US-7 ring form factor, with six different tissue layers (i.e., skin, fat, bone, artery, nerve, muscle) and metal electrodes with complex electrical characteristics defined for each material type. **b** Three electrode configurations that exhausts possible combinations of electrical current injection and voltage sensing electrodes used in 4-point bioimpedance sensing. **c**, **d** Volume impedance density (VID) surface plots for y-z (vertical cut of the artery from the middle point) and x-y planes (horizontal cut along the artery), respectively, where top plots and bottom plots show the VID with 22 mm and 2 mm electrode separation. In both cases, the primary artery VID contribution is higher with smaller electrode separation, shown as in brighter color. **e** Maximum values of the VID measured from the primary artery for different configurations (Config.), where the highest value obtained with Configuration 1 (pink) compared to Configurations 2 (green) and 3 (orange). **f** VID distribution along the primary artery for different electrode separations. The highest values are obtained with 2 mm electrode separation. Colors indicate the separation. **g** Percentage total impedance distribution between the primary (blue) and secondary (orange) arteries calculated from the volume integral of the VID measured per artery. In agreement with other analysis types, the highest specificity for the primary artery is achieved with 2 mm separation.

Bioimpedance sensing requires placement of a pair of injection and sensing electrodes responsible for stimulating the tissue with a high frequency AC signal and measuring the voltage difference that varies with the impedance variations in the underlying tissue composition. When sensors are placed around the finger, the measurements become sensitive to underlying hemodynamic activity (i.e., blood flow in digital arteries causing an expansion in artery diameter with the arrival of the blood pressure pulse wave) impacted by the electrode placement^23^. To assess such sensitivity profile and optimize the final bioimpedance ring design, we created human ring finger FEM with realistic physical dimensions of the underlying tissues accommodating two digital arteries branching from the palmer arch supplying blood to the finger^21^. The finger FEM is shown in Fig. 2a. We defined frequency-dependent electrical properties for each tissue type using Cole-Cole equations^24^ and ran electrical current simulations using AC/DC physics module of COMSOL Multiphysics v5.6.

A total of four electrodes (i.e., pairs of injection, and sensing) are required to initiate four-point (Kelvin) bioimpedance sensing. The most common arrangement of these electrodes is through placing them all along various (wrist, chest, ankle) arteries in a single line, with the outer electrodes used for the AC signal injection. On the other hand, in our case placing all four electrodes in a single line along the digital artery is not feasible due to the limited form factor of the ring. It is important to note that decreasing the electrode areas directly increases the electrode-to-skin impedance even at high frequencies and can significantly degrade the performance^25^. Therefore, we considered using 3 mm by 3 mm silver dry electrodes with only two electrodes sitting along the artery and the remaining two placed in parallel. Three main combinations arise when assigning the injection and sensing connections to the electrodes, as shown in Fig. 2b. For Configuration 1, the voltage sensing electrodes are fixed over the primary artery while the current injecting electrodes are spaced 2 mm apart. Configurations 2 and 3 both have the electrodes placed symmetrically centering the artery with a vertical distance: Configuration 2 has the voltage and sensing pairs diagonal from each other while Configuration 3 has the pairs across the artery from each other.

We simulated the whole geometry for each configuration at the operating frequency of 10 kHZ^13^ and measured the volume impedance density (VID) giving the local impedance contributions to the total bioimpedance using the principle of reciprocity^26,27^. VID along the artery provides the bioimpedance sensitivity (i.e., to the blood flow) and specificity (i.e., sensitivity distribution between the primary and the secondary artery) profiles (see Methods). Figure 2e shows the maximum values of VID measured along the primary artery comparing all three configurations. We observe that Configuration 1 provides the highest sensitivity among all three configurations, while Configuration 3 shows a significantly poor sensitivity, possibly due to the orthogonal placement of injection and sensing electrodes with respect to the underlying artery. Additionally, we tested the effect of physical separation between the injection and sensing electrode pairs with Configuration 1 through a sweep of distances (2 mm to 47 mm, with a 5 mm step), allowing the electrode set to go almost completely around the finger with a 55 mm diameter. For each separation, we captured the VID profiles (i) along the primary and secondary arteries, (ii) over the vertical slice in *y*–*z* plane cutting the geometry in middle at *x* axis, and (iii) over the horizontal slice in *x*–*y* plane cutting the geometry in the middle of the artery height at *z* axis, as shown in Fig. 2c, d. We observe that as the separation increases between the injection and sensing electrodes, VID from the primary artery initially decreases, with the lowest value obtained at 22 mm separation, and then starts increasing due to the injection electrodes circumnavigating the finger and getting closer to the sensing electrodes, with 2 mm separation providing the highest VID values from the primary artery (Fig. 2f). We also noted that as the separation increases the VID contribution from the primary artery with respect to the overall contribution from both arteries show a similar profile (i.e., initial decrease in percent VID contribution till 17 mm and increase till 47 mm), as shown in Fig. 2g. We concluded that 2 mm separation distance between injection and sensing electrodes provides the highest sensitivity to underlying artery, when sensing electrodes aligned with underlying the artery.

### Bioimpedance ring-sensor design, and experimental analysis

**Fig. 3:**
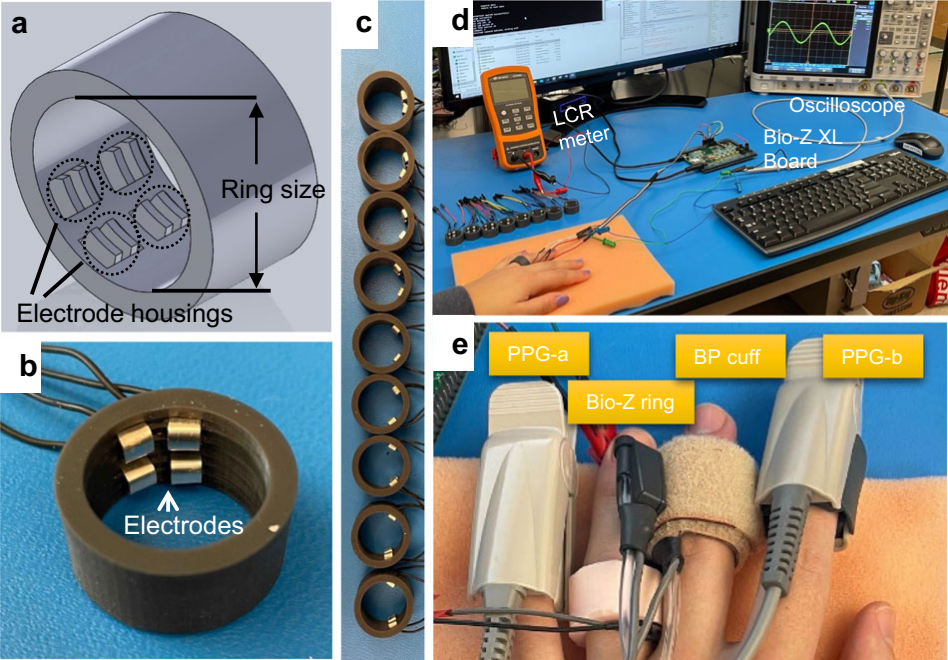
Bioimpedance ring-sensor. **a** 3-D SolidWorks design of the ring. **b** An example US-7 ring with 2 mm electrode separation. **c** A total of nine different electrode separation rings (2 mm to 18 mm, with 2 mm step). **d** Experimental setup used in assessing bioimpedance signal quality acquired with ring sensor. Bio-Z XL board is used for bioimpedance signal acquisition with data sent to PC for post-processing through a USB communication. The LCR meter is used to ensure good electrode-skin contact prior to experimentation. Oscilloscope is used to check the presence of saturation during the signal acquisition. **e** Different sensors on the same hand are used for blood pressure (BP) experiments. Bio-Z ring sensor is worn on the ring finger, with the Finapres NOVA BP cuff on the middle finger providing reference BP values. Photoplethysmography (PPG) sensors, PPG-a and PPG-b, are connected to the Bio-Z XL board and Finapres NOVA device respectively, and used for synchronization of BP beats with ring sensor bioimpedance features.

We designed and fabricated a total of 36 bioimpedance rings with nine different separations (2 mm to 18 mm, with a 2 mm step) and four different sizes (US 6.5 to 8, in 0.5 step sizes) as shown in Fig. 3a–c (see Methods for the design and fabrication process). The ring size used on each participant was chosen based on comfort and contact with the skin. Each ring includes four 3 mm by 3 mm silver electrodes, two of which are used for injecting a 10 kHz AC signal into the finger, and the remaining two are used to pick up the bioimpedance signals that change with the blood flow at the underlying digital artery. To assess the bioimpedance sensitivity to digital artery blood flow and verify the finite element simulations, we recruited *N* = *5* healthy individuals in their mid-twenties with institutional board approval (Methods). Initial to bioimpedance signal acquisition an LCR meter (Agilent U1731C, USA) reading is performed from the electrode pairs to ensure electrode-to-skin contact. All three configurations were tested on the first three participants using only 2 mm separation (due to its superior performance, and to avoid additional burden on the participants), while all five participants underwent the test involving different separations. During the experiments, the participants were asked to remain still with their left hand resting on a table, as shown in Fig. 3d. For each ring setup a total of five 30-second trials were conducted and the data is post-processed in MATLAB R2019b for sensitivity and signal fidelity analyses.

**Fig. 4:**
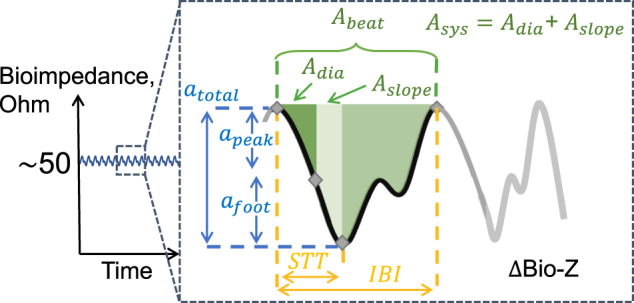
Bioimpedance signal illustration during a cardiac cycle and the corresponding bioimpedance feature definitions used in blood pressure correlation analysis. The amplitude features are shown in blue color, the timing features are shown in yellow color (STT: slope transit time, IBI: inter-beat intervals), and the area features are shown in green color. We named the feature pertaining to the area between the diastolic point and the maximum slope point as *A*_dia_, the area between the maximum slope point and the systolic point as *A*_slope_, and the area between the diastolic and the systolic point as *A*_sys_.

To assess the ring sensor sensitivity to digital artery blood flow, we analyzed the quasi-periodic bioimpedance signal with a characteristic morphology, as: (i) an impedance decrease from diastolic peak to systolic bottom with the increase in blood volume during systole, (ii) followed by an increase in the impedance as the blood volume leaves the sensing area, (iii) the second impedance decrease due the arrival of the reflection wave, (iv) a final increase in the impedance as the reflected blood volume leaves the sensing area, as shown in Fig. 4. As the participants were at rest, for every heartbeat we expect the morphology defined with the steps (i) to (iv) to change minimally, where any deviations are hypothesized to be the artifacts.

**Fig. 5:**
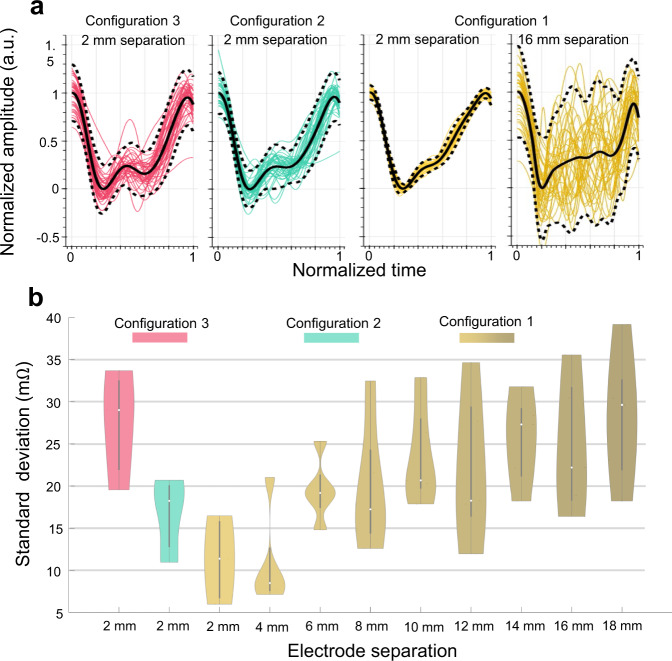
Bioimpedance signal consistency analysis results for different electrode configurations and sizes for data collected from *N* = *5* participants. **a** Bioimpedance signals over an example 30-seconds trial for different electrode configurations, visualizing the signal consistency over a constant period. The highest consistency is achieved with 2 mm ring separation using Configuration 1 (yellow) compared to Configuration 2 (green) and 3 (pink). **b** Violin plots for standard deviation (SD) metric used for signal consistency analysis calculated with different electrode configurations and separations. Violin plots include SD values averaged for all the trials of each participant and combined together. Box plots show the median and the Q25 and Q75 ranges. Colors used to identify separations and configurations. Here 2 mm and 4 mm electrode separations with Configuration 1 yield the lowest SD values indicating a higher overall bioimpedance signal consistency.

We initially normalized the time scale for each beat using the individual inter-beat intervals (IBI) to provide a fair comparison of morphological deviations under varying heart rates. We quantified the amount of deviation in beat-to-beat bioimpedance morphology using three different metrics: (1) standard deviation (SD) from the mean waveform morphology, (2) signal-to-noise ratio (SNR) from the fundamental heart rate frequency to the noise floor measured through the Fast Fourier Transform (FFT) of the full 30-seconds cycle, and (3) dynamic time warping (DTW) distance of waveforms to the mean waveform morphology for every beat (see Methods). We chose DTW distance as a metric to measure waveform similarities instead of Euclidean distance to account for morphological changes in waveforms from beat-to-beat due to the physiological reasons (e.g., blood flow rate impacting the timing of step (iii)–reflection wave arrival)^28^. Figure 5a provides an example for the ensembled bioimpedance beats for different ring separations and configurations with signals acquired from the same participant, where smaller ring electrode separation of Configuration 1 shows a more consistent waveform pattern compared to the case of higher separation or different configurations of injection and sensing electrode connections.

#### Assessment of blood-flow sensitivity based on injection and sensing electrode distributions

We observe the same performance trend with the FEM simulations for the performance associated with different configurations, where Configuration 1 yielded the highest average SNR, lowest average SD, and lowest average DTW distance against Configuration 2 and Configuration 3, as shown in Fig. 5b.

#### Assessment of blood-flow sensitivity at varying electrode spacing

The analysis of the effect of electrode separation when the injection electrodes aligned with the primary artery is shown in Fig. 5b for the metric SD, where the metrics SNR and DTW distance reveal similar performance for different separations. We observe a statistically significant decrease in performance with the increasing separation, in line with our findings from the finite element simulation, indicating the high sensitivity associated with smaller separation between injection and sensing electrode pairs.

### Bioimpedance and PPG waveform comparison on individuals with varying skin tones

**Fig. 6:**
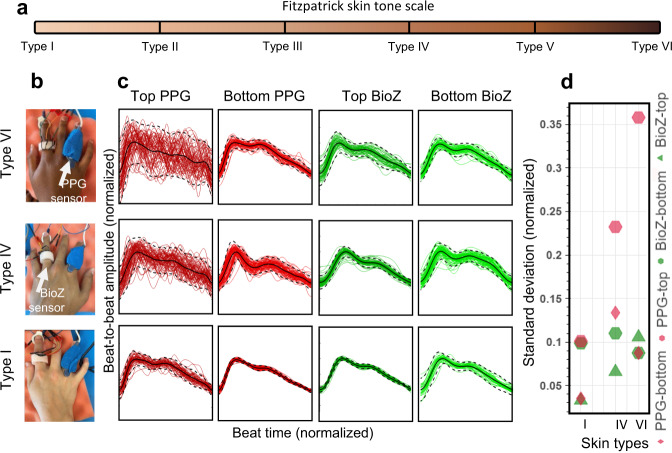
Comparison of arterial pulse wave captured with bioimpedance and PPG sensors for varying skin tones. **a** Fitzpatrick skin tone scale. In this study, we used Type I (pale white), Type IV (moderate brown), and Type VI (dark brown) skin types. **b** Bioimpedance (BioZ) ring sensor and Nellcor Maxfast PPG sensor (660 nm–red) placed at index and ring fingers respectively to capture simultaneous blood pressure pulse wave. During the measurements, the PPG sensor is wrapped around the finger with a self-adherent cohesive bandage to establish tight fit contact with the skin. **c** Normalized waveforms captured in two different scenarios placed on top of the skin, that is darker (plots at first and third columns) when compared to the placement on the bottom with a lighter skin tone (plots at second and fourth columns), from three participants with three skin types I, IV and VI. **d** Standard deviation values from the normalized beats with respect to the normalized average beat for different sensors and placements for different skin types. The results show a clear degradation in signal consistency for PPG sensor when the skin color gets darker, whereas no significant correlation was observed for bioimpedance sensor.

Commercial wearable ring sensors (e.g., Oura Ring) have been recently made available that rely on optical, PPG modality. However, there are studies that show the sensitivity of PPG signal quality in presence of varying skin tones^29,30^, whereas bioimpedance does not discriminate as it pertains to skin tones. This limits the effective use of PPG for capturing blood volume changes for participants with darker skin tones. For this reason, we carried out an analysis to compare the two modalities: given that the PPG sensor used in our study is from a finger clip rather than a sensor that can be placed within a ring form factor, and to conduct a like-for-like comparison between bioimpedance and PPG, especially for participants with darker skin tones, we executed additional analyses. We used an adhesive Nellcor Maxfast PPG sensor (660 nm—red) and our bioimpedance ring (see Fig. 6b), where we collected simultaneous bioimpedance and PPG signals from the base of the ring and index fingers, for three participants with different skin tones (Fitzpatrick skin tone scaling^31^: Type I—pale white, Type IV—moderate brown, and Type VI—dark brown). The Nellcor sensor placement resembles what we would expect from a ring form factor, where prior to the measurements the sensor is wrapped around the finger with a self-adherent cohesive bandage to establish tight fit contact with the skin. Initially, we placed the sensors at the bottom of the finger which has a lighter skin tone compared to the top of the finger. Next, we placed the sensors on top of the finger. We observed a clear degradation of the beat-to-beat consistency for PPG in Type VI skin-toned participants (standard deviation from normalized mean waveform morphology increased from 0.09 to 0.36 from bottom to top), whereas no significant difference was observed for bioimpedance with varying skin tones (see Fig. 6c, d).

### Ring-sensor blood pressure estimations

The ring-sensor and BP data were collected from *N* = *10* presumably healthy male participants in their mid-twenties. Each participant wore a bioimpedance ring-sensor (Configuration 1 with 2 mm separation) at their ring finger, with participants’ control BP measurements coming from a medical-grade BP monitoring finger cuff device, Finapres NOVA, placed at the middle finger participant along with a standard brachial arm cuff for initial calibrations, as shown in Fig. 3e (Methods). A handheld ultrasound device is used for precise artery localization for optimal placement of the bioimpedance sensor.

To achieve a dataset of fluctuating systolic and diastolic BPs, participants were asked to perform periods of cold pressor exercises, which is the process of increasing BP by placing the individual’s non-sensor (right) hand in an ice bath for a time period of 1–2 minutes (based off their comfort). The cold pressor exercise induces higher BPs within an individual by activating the sympathetic nervous system to increase cardiac output and constrict the peripheral arteries^32^. In addition, periods of rest were collected in between the cold pressor exercises to capture the participant’s return to their baseline BP before starting the next iteration of cold pressor exercise. Each participant was asked to repeat this process of cold pressor and rest for at least four iterations. More than ten thousand BP datapoints were captured across all participants along with corresponding continuous bioimpedance signal from the ring.

#### Blood pressure model training and performance evaluation

**Fig. 7:**
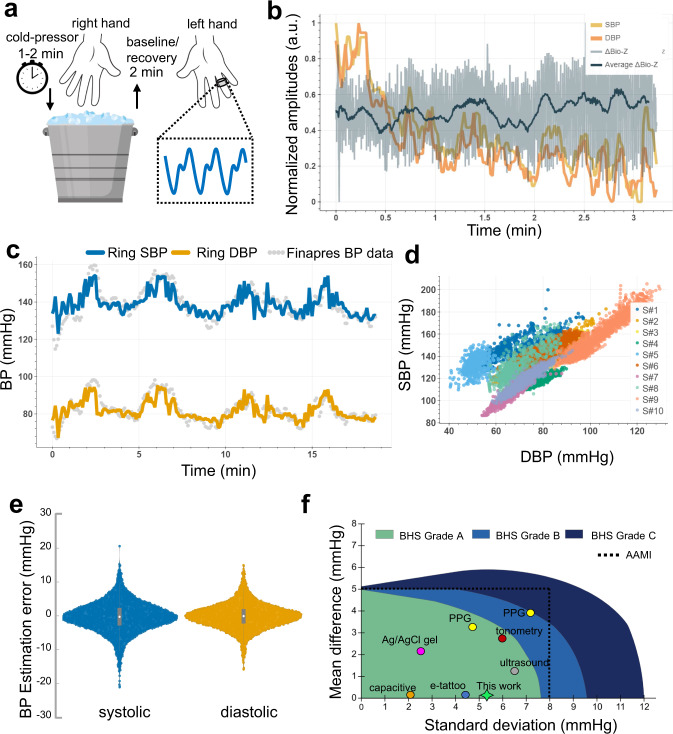
Bioimpedance ring sensor systolic (SBP) and diastolic (DBP) blood pressure results. **a** Visualization of cold-pressor test to induce blood pressure variations. **b** SBP (yellow line) and DBP (orange line) beats, and corresponding bioimpedance signal (gray line) and its mean (dark blue line) for a single trial collected at the participant’s recovery from hand-grip/cold-pressor test. **c** SBP (blue line) and DBP (orange line) estimations with bioimpedance ring sensor in reference to gold-standard Finapres finger BP cuff measurements (gray circles) for a single participant (S#3). **d** SBP and DBP value distributions for all participants, showing the wide range of blood pressure values used in evaluation. Colors represent data from different participants. **e** Violin plots for SBP (blue) and DBP (orange) estimations errors with ring sensors in comparison to reference Finapres BP. The gray box plot shows the median, and 25th and 75th quartiles. **f** Comparison of related work with this work for cuffless BP estimation and their classification based on the AAMI and BHS standards. Green, light blue, and dark blue areas represent Grade A, Grade B, and Grade C classifications for the BHS standard. Dashed black line represents the error limits set by the AAMI standard.

The cold pressor test allowed to achieve a wide diversity of systolic and diastolic BP ranges of 89–213 mmHg and 42–122 mmHg, respectively, indicating the success of maneuvers in fluctuating continuous BP (Fig. 7a–d). These high BP ranges enabled to test our system performance at hypertensive scale (i.e., systolic >140 mmHg, and diastolic >90 mmHg), although all the participants were normotensive. The cold-pressor-induced systolic and diastolic BP patterns estimated with the bioimpedance ring sensors for one participant are shown in Fig. 7c. Supplementary Tables 2–3 show the mean errors (ME) and standard deviations (SD), as well as the correlation for bioimpedance ring-sensor estimations of SBP and DBP, for all ten participants. Figure 7e shows violin plots based on the estimation errors for SBP and DBP with data combined from all participants. The bioimpedance ring sensors achieved an estimation performance on average 0.11 ± 5.27 mmHg and 0.11 ± 3.87 mmHg (mean error ± standard deviation) for SBP and DBP, respectively with less than 6 mmHg root-mean-squared error values. These statistical errors are well within the range based on the requirements set by the AAMI standard^22,33^. In addition, we achieve high correlations (Pearson’s correlation coefficient, on average 0.76 and 0.81 for SBP and DBP respectively), indicating the use of a bioimpedance ring as a convenient, compact device with strong BP estimation ability.

Collected reference BP and bioimpedance signals were synchronized based off their interbeat intervals for each waveform cycle to ensure perfect alignment of bioimpedance features with corresponding beat-to-beat BP. Each waveform cycle is analyzed to extract key features in estimating BP. These features can be broken down into four main categories which include: amplitude values, time intervals, area under waveform, and gradients calculated from the derivative of the Bio-Z waveform. In total 15 extracted bioimpedance waveforms features were utilized. Both feature set and BP values were applied a 10-beat window moving average filter with 50% overlap to reduce measurement noise and interbeat variability. We created two separate machine-learning models for systolic and diastolic BP using an AdaBoost regression model. The model was tuned to have 50 estimators with a tree depth of 5. For each participant separate models were created using a K-fold (*K* = 5) for cross validation, to calculate estimation performance across the complete measurement duration. Once all data points were predicted statistical errors including the mean error, standard deviation, and root mean squared error were calculated for each subject and both BPs. Additionally, the Pearson correlation coefficient is reported to highlight the strong linearity between predicted and actual BPs within an expanded range (see Methods for additional details for feature extraction and model training).

**Fig. 8:**
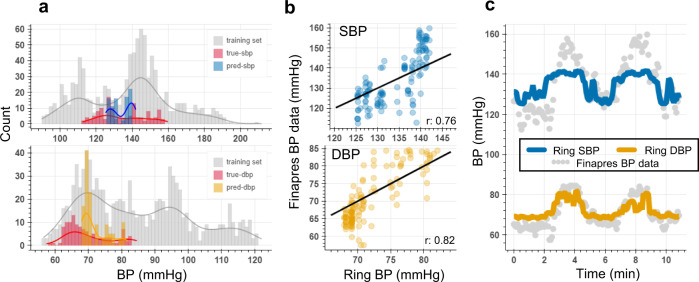
Bioimpedance ring sensor leave-one-subject-out analysis for systolic (SBP) and diastolic (DBP) blood pressure estimation. **a** SBP (top) and DBP (bottom) data distribution training set involving participants S#6, S#7, and S#9 shown in gray color, true SBP, and DBP values in the test set for S#8’s’data shown in red color, and predicted SBP (blue) and DBP (orange) values for S#8’s data. **b** Correlation plots for SBP and DBP between true and predicted BP points, showing high correlation values (*r* represents Pearson’s correlation coefficient between true and predicted BP values). **c** Time traces for SBP (blue line) and DBP (orange line) estimations with bioimpedance ring sensor for S#8, and the reference BP values (gray circles), where the model was trained on three other participants’ data.

An additional preliminary analysis has been conducted to assess the generalizability of our BP estimation model using bioimpedance ring sensor. A leave-one-subject-out paradigm for a single participant (S#8) with their BP range (systolic BP: 112–160 mmHg, diastolic BP: 57–85 mmHg) being within the group BP range (systolic BP: 89–213, diastolic BP: 42–122) is included. We used the data from three participants and tested the model performance on a separate participant with a single value calibration measurement to account for the offset, where the last systolic and diastolic BP values are used as a calibrating factor. This analysis shows promising results with peak correlations of 0.76/0.82 (between estimated and true systolic/diastolic BP), offering great potential for construction of generalizable models (see Fig. 8 and Supplementary Table 4). An extension of the leave-one-subject-out analysis over all ten participants is provided in Supplementary Note 1 and Supplementary Table 5.

## Discussion

Our contribution in this paper is summarized as follows: (i) We present an end-to-end framework for a wearable blood flow and blood pressure monitoring system in the form of a ring-shaped device that depends on non-invasive bioimpedance modality. (ii) We base the unique design of the bioimpedance ring sensor on a custom-designed finite element model of the human finger. This allows us to test a variety of factors involved in bioimpedance sensing, including the optimization of electrode-skin contact points. The proper placement of the electrodes and their functionality assignment as AC signal injection or voltage sensing significantly impacts the bioimpedance signal sensitivity to the underlying blood flow. This becomes particularly important given that the rings have a limited active sensing area to host the sensors, when compared to other wearable form factors. (iii) We designed and fabricated a total of 36 individual rings with different size and separations and performed exhaustive experiments to verify the finite element model simulation observations. (iv) We provided an analysis demonstrating high consistency for the signals acquired with bioimpedance sensor when compared to PPG sensors for participants with darker skin tones. (v) We provide a proof-of-concept study with the bioimpedance ring sensors in estimating systolic and diastolic blood pressure values with a performance well within the ranges recommended by the AAMI standard^22^. Figure 7f shows the reported performance of existing work (based on PPG^34^, ultrasound^35^, tonometry^36^, capacitive sensor^37^, e-tattoo bioimpedance^38^, Ag/AgCl gel bioimpedance^39^, See Supplementary Table 1) on cuffless blood pressure monitoring and their classification within the AAMI standard and British Hypertensive Society (BHS) accuracy grading categories (Grades A, B, and C)^22^. Our analysis over a large number of validation points (~140 minutes of measurements, yielding on average ~845 ± 380 seconds of continuous BP data recorded per participant, and 10,145 data points used for evaluation) and with varied systolic and diastolic BP ranges shows a strong potential in replacing the conventional paradigm of how we measure blood pressure.

The bioimpedance ring sensors provide a unique opportunity to non-invasively capture important hemodynamic parameters, while offering significant advantages over the current medical diagnostic tools, such as traditional sphygmomanometer that uses an obtrusive cuff. Also, the ring sensor ensures a firm electrode-to-skin contact due to the tight wear of the ring. Hence, the sensor measurements provide consistent readings of the bioimpedance at the same location over a long period of time, unlike other research level wearable systems that are designed for various limbs (e.g., wrist, ankle, chest) where these sensors are exposed to loss-of-contact during any movement. The primary challenge with ring sensors is the limited active area that the measurements can take place due to smaller dimensions of the human finger in comparison to other human limbs. Therefore, we provided a thorough simulation and design of the ring-sensor, to ensure high-sensitivity operation. Our exhaustive experimental analysis provides a proof-of-concept demonstration for the ring sensor to be used in capturing the blood volumetric changes at the digital arteries. In addition, although we observed an offset in quantitative analysis between simulations and experiments, the general trends match with the experimental results, indicating the significance of proposed simulation framework. We provide an analysis for the ring sensors in capturing unique features from the bioimpedance signal and build regression models to estimate SBP and DBP, showing the potential of this framework to improve the traditional diagnostic tools used for medical practice. Although this study had a very specific focus on arterial blood pressure tracking, in the future, several directions including the use of our bioimpedance ring for additional applications (e.g., body tissue composition^40^) could be explored.

## Methods

### FE model design and VID estimation

An anatomical cross-sectional representation of human finger is used as the basis of the finger finite element model. The representation is refined to smooth out sharp edges and simplify the components into six separate tissue layers: skin, fat, muscle/tendon, bone, artery, nerve. It is then imported into COMSOL Multiphysics v5.6, converted into a work plane, and scaled to a size US-7 ring diameter, matching the experimental studies conducted, and extruded 2 cm into a 3D geometry. The domains of the model are assigned as the Skin, Tendons/ligaments, Blood, Muscle, and Fat. Each domain uses Cole-Cole model parameters to assign its electrical conductivity and permittivity. A four 3 mm by 3 mm silver electrodes is attached to the geometry. The spacing between these electrodes is designed parametrically to allow for parametric sweeps of separation distance. The model was meshed using Physics-controlled Fine mesh. The AC/DC physics module is used to inject a current through the injection electrodes.

For VID analysis, another AC/DC physics module is defined for the sensing electrodes, where VID is calculated using the principle of reciprocity as in Eq. (1).

1 $${\it{VID}} = \rho \frac{{\overrightarrow {J_v} \cdot \overrightarrow {J_s} }}{{I^2}}$$

Here, $\rho$ is the blood resistivity, $\overrightarrow {J_v}$ and $\overrightarrow {J_s}$, are the current density vectors associated with the individual injection from voltage sensing and current injection electrodes, where their vectoral dot-product provides the measure of sensitivity, $I$ is the amplitude of the injected current which is set to 1 A for simplicity. VID values for the primary and secondary artery as well as the total model are obtained using a domain integration function. For arteries VID is measured along a 1-dimensional line running through the center of the artery.

### Ring-sensor design and fabrication

Four different sizes of nine different separation rings were designed in SolidWorks (2020) and 3D printed using a 3D printer (Form 3+, Formlabs, USA) in a flexible filament. The inner diameter was sized according to the individual subject’s left ring finger to ensure a good connection between the electrodes and the skin. The rings have a thickness of 2.5 mm to prevent tearing of the flexible filament. Four 3 mm × 3 mm silver electrodes were placed in two rows on the inner curve of the ring. Three extruded cuts were made, where the electrodes would be placed along with an extruded base between the outer two extruded cuts allowing the electrodes to be raised 1 mm. The raised base was designed so that the electrodes would make better contact with the skin and decrease the skin-electrode impedance. The extruded cut in the center of the raised base allowed the soldered wire to be pulled directly through the ring. The 3 mm × 3 mm electrodes were fabricated by cutting the strips of silver to 8 mm and length and bending the silver over a 3D printed 3 mm × 3 mm rectangular rod.

### Bioimpedance signal acquisition and post-processing

A custom developed printed-circuit board (PCB), called Bio-Z XL-board, was used to run bioimpedance experiments. The Bio-Z XL-board uses an ARM Cortex M4 microcontroller (MCU) that operates a 16-bit digital-to-analog converter (DAC, Texas Instruments) and a 24-bit analog-to-digital converted (ADC, Texas Instruments, 0.3-μV resolution), to inject a programmable frequency and gain AC signal and sample (with 93,750 Hz sampling frequency) the voltage signal that is picked-up with voltage sensing electrodes, high-pass filtered and amplified with an instrumentational amplifier (IA, Analog Devices), respectively. The sampled signal is then sent to a PC with high-speed USB communication. A series I-Q demodulation is held to obtain the changes in the bioimpedance signal. The demodulated signal is then filtered with 2nd order Butterworth band-pass filter (frequencies, low-cutoff: 0.05 Hz, high-cutoff: 6 Hz) to capture the changes in bioimpedance due to the pulsatile quasiperiodic blood flow and mitigate out-of-frequency band noise.

### Experiments with human participants

All the experiments with the human participants were performed under the approval of the Institutional Review Board of Texas A&M University (IRB no. IRB2017-0086D), where all participants provided written informed consent to take part in the experiments. A total of *N* = 16 participants, with no known health conditions participated in this study. Five out of 16 participants (4/1 female/male, age range/median: 21–22/22 years) were involved in the experiments for ring electrode optimization, wearing rings with different configurations and separations of electrodes at sitting position, with their hand resting at an adjustable table. 10 out of 16 participants (1/9 female/male, age: range/median:19–26/21 years) were involved in the bioimpedance ring to BP correlation study, wearing a bioimpedance ring of Configuration 1 with 2 mm separation between injection and sensing electrodes on ring finger of left hand, a finger cuff in middle finger for continuous beat-to-beat SBP and DBP readings that are calibrated with prior brachial cuff readings, two PPG devices on the little and index fingers used for synchronizing cuff BP sensor with bioimpedance acquisition board. The participants were asked to do a 1 to 2 min cold pressor test with their right hand to cause an elevation in their BP values, followed by a rest period to capture baseline recovery of BP. 3 out of 16 participants (2/3 female/male, age: range/median: 20–23/22) involved in skin-tone analysis, where participants were asked to wear Nellcor Maxfast PPG sensor and bioimpedance ring sensor on their index and ring fingers.

### Beat-by-beat bioimpedance signal coherence and fidelity analysis

The per-beat bioimpedance signals obtained from each cardiac cycle are ensembled together based on their start and end points after a time normalization based on the per-cycle IBI values. Additionally, for morphological resemblance analysis, each signal is normalized in amplitude, with the diastolic impedance peak placed at the origin. For each trial, a mean signal waveform is calculated using all ensembled signals. Each ensembled signal is then compared to the group mean based on three quality metrics: standard deviation (SD), signal-to-noise ratio (SNR), and dynamic time-warping (DTW) distance. For SD, standard deviation of each data point from within the cycle from the group mean was calculated and averaged to obtain the final SD value. To calculate the SNR, the Fast Fourier Transform (FFT) of the full 30-seconds trial is obtained in MATLAB, the signal power at the fundamental frequency that is representative of the quasi-periodic heart rate activity, with respect to the noise level is measured based on the FFT magnitudes. Lastly, the DTW distances were calculated using built-in DTW function of MATLAB with every cycle compared to the group mean. Once again, the DTW values were averaged for each trial to get the final DTW distance value.

### Bioimpedance signal feature extraction

The main types of features are extracted from each per-beat bioimpedance cycle, these are: amplitude, area, and time features. Amplitude features take the difference in amplitudes from characteristic points within the signal. The peak and foot amplitudes are calculated by taking the difference in amplitudes from the diastolic and systolic points respectively to the maximum slope point. Additionally, the total $\Delta$Bio-Z amplitude is the difference from the diastolic to systolic points of the signal. Area features calculate the area under the bioimpedance curve in between two characteristic points. These features have shown to have high correlation with total peripheral resistance, key to estimating blood pressure. Time features provide a proxy to the pulse wave velocity as well as the duration of the systolic and diastolic phases of each cardiac cycle.

### BP correlation analysis

The reference beat-to-beat BP measurements obtained with the Finapres NOVA finger cuff and continuous bioimpedance ring sensor readings are synchronized in time using the IBI information captured from finger PPG measurements obtained at two fingers of the left hand. Pearson’s correlation coefficients were calculated, and reported per subject for each bioimpedance feature with systolic and diastolic BP. The change in bioimpedance signal (∆Bio-Z), when the sensor is placed along the artery represents the volumetric changes due to pulsatile blood flow. Specifically, the decrease in impedance is representative of the blood arrival through the sensing area, this is also known as the systolic phase when the left ventricle of the heart beats to push blood through the arteries. Likewise, the phase of increased impedance is known as the diastolic phase when the heart rests between beats. The volumetric change ($dV$) with every cardiac cycle is correlated to the pressure exerted on the walls of the artery, namely blood pressure (BP) with Eq. (2)^41^.

2 $${\it{dV}} = {\it{C}}_0e^{ - \alpha P}{\it{dP}}$$

Here, $P$ and $C_0$ represent the pressure and artery compliance at rest, respectively, and $\alpha$ represents the degree of BP dependence, which is shown to be a factor of the pressure due to the pressure-dependent elasticity of the artery walls^42^.

Certain characteristic features extracted from non-invasive bioimpedance signal captured at the digital arteries through the ring sensor carries volumetric and hemodynamic information related to the aforementioned dynamics of blood flow, and hence correlates with the SBP and DBP for each cardiac cycle. These features can be broken down into four main categories which includes: amplitude values, time intervals, area under waveform, and gradients calculated from the derivative of the Bio-Z waveform. In total 15 extracted bioimpedance waveforms features (three amplitude features: *peak*, *foot* and *inflection point* (*ip*), seven time interval features: slope transit time, *peak*-to-*maximum slope point* (*msp*), *msp*-to-*foot*, *peak*-to-*ip*, *foot*-to-*ip*, *msp*-to-*ip*, total beat duration, two gradient features: $(\delta Z/\delta t)_{msp}$, $(\delta Z/\delta t)_{ip}$, three area features: *peak*-to-*foot*, *foot*-to-next cycle *peak*, total beat area) were utilized. Both feature set and BP values were applied a 10-beat window moving average filter with 50% overlap to reduce measurement noise and interbeat variability. Two separate machine learning models for systolic and diastolic BP using an AdaBoost regression model was build. The model hyperparameters were tuned to have 50 estimators with a tree depth of 5. The data is shuffled using scikit-learn library shuffle function in Python 3.8. prior to train/test split to ensure model learning at different BP ranges. For each participant separate models were created using a fiveold cross-validation, to calculate estimation performance across the complete measurement duration. Once all data points were predicted statistical errors including the mean error, standard deviation, and root mean squared error were calculated for each subject and both BPs. Additionally, the Pearson correlation coefficient is reported to highlight the strong linearity between predicted and actual BPs within an expanded range.

### Reporting summary

Further information on research design is available in the Nature Research Reporting Summary linked to this article.

## Supplementary information

Supplementary Information

REPORTING SUMMARY

## Data Availability

The dataset supporting the findings of this study is available on GitHub at https://github.com/TAMU-ESP/Ring_BP_Python under the file name *Feature_and_BP_Subject_Data.mat*. The associated preprocessed raw data are available and can be shared with interested parties upon reasonable request.

## Code availability

The machine learning algorithms are publicly available on GitHub at https://github.com/TAMU-ESP/Ring_BP_Python. The custom codes used for data visualization are available from the corresponding authors upon request.
