# Supplementary material for: Continuous cuffless blood pressure monitoring with a wearable ring bioimpedance device
Source: NPJ Digit Med. 2023 Mar 30;6:59. doi: 10.1038/s41746-023-00796-w (PMC10063561; doi:10.1038/s41746-023-00796-w)
Supplement: Supplementary file 1 — Supplementary Information [file 41746_2023_796_MOESM1_ESM.pdf]

SUPPLEMENTARY INFORMATION

for

Continuous Cuffless Blood Pressure Monitoring with a Wearable Ring Bioimpedance Device

Kaan Sel[1], Deen Osman[1], Noah Huerta[2], Arabella Edgar[3], Roderick I. Pettigrew[4], and Roozbeh Jafari[1,3,4,5]*

[1]Department of Electrical and Computer Engineering, Texas A&M University, College Station, TX, USA

[2]Department of Mechanical Engineering, Texas A&M University, College Station, TX, USA

[3]Department of Biomedical Engineering, Texas A&M University, College Station, TX, USA

[4]School of Engineering Medicine, Texas A&M University, Houston, TX, USA

[5]Department of Computer Science and Engineering, Texas A&M University, College Station, TX, USA

*corresponding author: rjafari@tamu.edu

Supplementary Table 1

Supplementary Table 1. Comparison of cuffless blood pressure (BP) technologies. *Mean radial BP; † Arterial BP, based on 3 single static measurements;

Cuffless BP Technology	Systolic BP, mmHg		Diastolic BP, mmHg	
	**Mean difference**	**Standard deviation**	**Mean difference**	**Standard deviation**
Tonometry[1]	-0.7	6.6	2.7	5.8
Ultrasound[2]	-1.3 (MD) ± 6.5 (SD)*			
PPG[3]	Vary, 3 to 15		Vary, 3 to 12	
Capacitive pressure sensor[4]	-0.1 (MD) ± 2.1 (SD)			
Bioimpedance wrist sensor (Ag/AgCl)[5]	2.5	3.4	1.9	2.6
Bioimpedance wrist sensor (graphene)[6]	0.2	5.8	0.2	4.5
**Bioimpedance ring sensor (this work)**	**0.1**	**5.3**	**0.1**	**3.9**

Supplementary Table 2

Supplementary Table 2. Bioimpedance ring sensor systolic blood pressure (SBP) estimation results.

	Systolic BP, mmHg							
**Participant ID**	**Mean difference**	**Standard deviation**	**Correlation**	**RMSE**	**BP min-max (range)**	**BP mean**	**BP standard deviation**	**N**
S#1	-0.02	8.92	0.49	8.92	130-196 (149)	149	10	136
S#2	1.09	7.18	0.82	7.26	119-171 (52)	144	12	89
S#3	-0.20	5.67	0.73	5.68	115-160 (45)	139	8	225
S#4	-0.06	3.55	0.55	3.56	106-126 (20)	116	4	162
S#5	-0.19	3.75	0.49	3.75	120-142 (22)	133	4	126
S#6	0.09	5.69	0.85	5.70	118-161 (43)	141	11	342
S#7	0.10	3.28	0.92	3.28	89-127 (38)	109	8	295
S#8	0.18	4.71	0.93	4.72	112-160 (47)	135	13	131
S#9	0.21	5.75	0.94	5.75	135-213 (77)	157	16	371
S#10	-0.10	4.16	0.88	4.16	101-139 (38)	119	9	165
**Average**	**0.11**	**5.27**	**0.76**	**5.28**	**115-159 (44)**	**134.2**	**9.6**	**204.2**

Supplementary Table 3

Supplementary Table 3. Bioimpedance ring sensor diastolic blood pressure (DBP) estimation results.

	Diastolic BP, mmHg							
Participant ID	Mean difference	Standard deviation	Correlation	RMSE	BP min-max (range)	BP mean	BP standard deviation	N
S#1	0.38	6.12	0.70	6.13	55-95 (40)	70	9	136
S#2	0.59	5.23	0.83	5.26	60-100 (40)	82	9	89
S#3	0.22	4.33	0.74	4.34	67-98 (31)	82	6	225
S#4	-0.37	3.55	0.54	3.57	64-87 (23)	77	4	162
S#5	0.37	4.00	0.92	4.02	42-77 (34)	58	10	126
S#6	-0.07	4.32	0.80	4.32	63-96 (34)	79	7	342
S#7	0.00	2.35	0.86	2.35	56-78 (22)	68	5	295
S#8	-0.12	2.60	0.94	2.60	57-85 (27)	71	7	131
S#9	-0.06	3.48	0.93	3.48	87-122 (35)	103	9	371
S#10	0.00	2.70	0.87	2.70	61-88 (27)	73	6	165
**Average**	**0.11**	**3.87**	**0.81**	**3.88**	**61-93 (32)**	**85.1**	**8.42**	**204.2**

Supplementary Table 4

Supplementary Table 4. Leave-one-subject-out analysis with bioimpedance ring sensor for systolic diastolic blood pressure (BP) estimation.

	Systolic BP, mmHg				Diastolic BP, mmHg			
Experiment type (subject ID)	Mean difference	Standard deviation	Correlation	RMSE	Mean difference	Standard deviation	Correlation	RMSE
Leave-one-subject-out (S#8)	-0.62	9.11	0.76	9.13	1.23	4.62	0.82	4.78

Supplementary Table 5

Supplementary Table 5. Leave-one-subject-out analysis for all subjects based on a deep neural network model estimating systolic and diastolic BP changes based on the changes in features extracted from beat-to-beat bioimpedance signal. For each participant, the first and last reference BP points are used to calibrate for the BP offset. *The model for S#9 is trained with the additional one-third of S#9's data and tested on the remaining two-thirds of S#9's data due to the higher BP range of S#9 compared to the rest of the participants. This higher range in S9 is not available in other participants and hence the model cannot effectively learn how to estimate such unforeseen data points.

	**Systolic BP, mmHg**		**Diastolic BP, mmHg**	
**Participant ID**	**Mean difference**	**Standard deviation**	**Mean difference**	**Standard deviation**
S#1	5.74	10.12	0.27	10.48
S#2	10.77	11.67	7.14	8.61
S#3	13.32	9.55	6.70	6.30
S#4	1.97	4.22	3.49	4.10
S#5	6.34	5.60	4.71	8.42
S#6	2.36	10.04	1.09	7.05
S#7	3.13	7.62	1.35	4.25
S#8	7.90	12.03	3.93	6.22
S#9*	2.71	10.97	0.72	6.84
S#10	0.65	10.69	0.27	6.42
**Average**	**5.49**	**9.25**	**2.97**	**6.87**

Supplementary Note 1

We provide an additional analysis with leave-one-subject-out type of evaluation for all 10 participants. For this analysis, we created deep neural network models consisting of three fully connected network layers (number of neurons: 32, 8, 3), with the last layer having three output values that includes SBP, DBP and MAP. The models are optimized with the Adam Optimizer (learning rate: 2e-4, beta-1: 0.5). We trained the models using the changes of the feature values extracted from the bioimpedance signal and changes in the reference SBP, DBP, and MAP, with data obtained from nine participants, and tested with the bioimpedance data obtained from the participant that is excluded from the model training. The model estimations provide changes in SBP and DBP, where we used the average of the offset from the first and last reference BP values to calibrate the model estimations and obtain the final prediction values of SBP and DBP. We apply a 10-beat moving average with 50% overlap to the estimations to reduce measurement noise and interbeat variability. This method has been repeated for every participant except S#9, since their BP range was significantly higher than the rest of the group (see Supplementary Table 2, max/mean SBP and DBP for S#9: 213/157 mmHg and 122/103 mmHg, respectively; group max/mean SBP and DBP excluding S#9: 196/132 mmHg and 100/83 mmHg, respectively), where the range of BP (SBP: 196-213 mmHg, DBP: 100-122 mmHg) in S#9 is not present in the other participants' data. Therefore, for S#9, testing the model that is trained based on leave-one-subject-out settings leads to an unfair assessment, as the model cannot effectively learn how to estimate such unforeseen data points. To address this mismatch, during the model training for S#9, we included the first one-third of S#9's data in the training set along with the data from the other nine participants and tested the model performance on the remaining two-thirds of S#9's data. The results of this analysis are shared in Supplementary Table 5.

Supplementary References

1. Kemmotsu, O. et al. Blood pressure measurement by arterial tonometry in controlled hypotension. Anesth Analg 73, 54–58 (1991).
2. Soleimani, E., Mokhtari-Dizaji, M., Fatouraee, N. & Saberi, H. Assessing the blood pressure waveform of the carotid artery using an ultrasound image processing method. Ultrasonography 36, 144 (2017).
3. Yang, S., Zhang, Y., Cho, S.Y., Correia, R. & Morgan, S. P. Non-invasive cuff-less blood pressure estimation using a hybrid deep learning model. Opt Quantum Electron 53, 1–20 (2021).
4. Kim, J. et al. Soft wearable pressure sensors for beat-to-beat blood pressure monitoring. Adv Healthc Mater 8, 1900109 (2019).
5. Ibrahim, B. & Jafari, R. Cuffless Blood Pressure Monitoring from an Array of Wrist Bio-impedance Sensors using Subject-Specific Regression Models: Proof of Concept. IEEE Trans Biomed Circuits Syst (2019) doi:10.1109/TBCAS.2019.2946661.
6. Kireev, D. et al. Continuous cuffless monitoring of arterial blood pressure via graphene bioimpedance tattoos. Nat Nanotechnol 1–7 (2022).
