# Supplementary material for: Continuous cuffless blood pressure monitoring with a wearable ring bioimpedance device
Source: NPJ Digit Med. 2023 Mar 30;6:59. doi: 10.1038/s41746-023-00796-w (PMC10063561; doi:10.1038/s41746-023-00796-w)
Supplement: Supplementary file 2 — REPORTING SUMMARY [file 41746_2023_796_MOESM2_ESM.pdf]

nature portfolio

Corresponding author(s): Roozbeh Jafari

Last updated by author(s): 02/25/2023

Reporting Summary

Nature Portfolio wishes to improve the reproducibility of the work that we publish. This form provides structure for consistency and transparency in reporting. For further information on Nature Portfolio policies, see our Editorial Policies and the Editorial Policy Checklist.

Statistics

For all statistical analyses, confirm that the following items are present in the figure legend, table legend, main text, or Methods section.

n/a	Confirmed	
☐	☒	The exact sample size (*n*) for each experimental group/condition, given as a discrete number and unit of measurement
☐	☒	A statement on whether measurements were taken from distinct samples or whether the same sample was measured repeatedly
☒	☐	The statistical test(s) used AND whether they are one- or two-sided<br>*Only common tests should be described solely by name; describe more complex techniques in the Methods section.*
☒	☐	A description of all covariates tested
☒	☐	A description of any assumptions or corrections, such as tests of normality and adjustment for multiple comparisons
☐	☒	A full description of the statistical parameters including central tendency (e.g. means) or other basic estimates (e.g. regression coefficient) AND variation (e.g. standard deviation) or associated estimates of uncertainty (e.g. confidence intervals)
☒	☐	For null hypothesis testing, the test statistic (e.g. *F*, *t*, *r*) with confidence intervals, effect sizes, degrees of freedom and *P* value noted<br>*Give P values as exact values whenever suitable.*
☒	☐	For Bayesian analysis, information on the choice of priors and Markov chain Monte Carlo settings
☒	☐	For hierarchical and complex designs, identification of the appropriate level for tests and full reporting of outcomes
☐	☒	Estimates of effect sizes (e.g. Cohen's *d*, Pearson's *r*), indicating how they were calculated

Our web collection on statistics for biologists contains articles on many of the points above.

Software and code

Policy information about availability of computer code

Data collection	Finapres® NOVA, Visual Studio, C++, COMSOL Multiphysics, SolidWorks
Data analysis	Python 3, MATLAB

For manuscripts utilizing custom algorithms or software that are central to the research but not yet described in published literature, software must be made available to editors and reviewers. We strongly encourage code deposition in a community repository (e.g. GitHub). See the Nature Portfolio guidelines for submitting code & software for further information.

Data

Policy information about availability of data

All manuscripts must include a data availability statement. This statement should provide the following information, where applicable:

- Accession codes, unique identifiers, or web links for publicly available datasets
- A description of any restrictions on data availability
- For clinical datasets or third party data, please ensure that the statement adheres to our policy

The dataset supporting the findings of this study is available on GitHub at https://github.com/TAMU-ESP/Ring_BP_Python under the file name Feature_and_BP_Subject_Data.mat. The associated preprocessed raw data are available and can be shared with interested parties upon reasonable request.

Human research participants

Policy information about studies involving human research participants and Sex and Gender in Research.

Reporting on sex and gender	Sex or gender is not considered as a factor in the study design.
Population characteristics	A total of N=16 presumably healthy participants, ages 19 to 26, participated in this work with N=9 male an N=7 female.
Recruitment	The recruitment was performed in full accordance to the IRB protocol.
Ethics oversight	Institutional Review Board of the Texas A&M University (IRB no. IRB2017-0086D).

Note that full information on the approval of the study protocol must also be provided in the manuscript.

Field-specific reporting

Please select the one below that is the best fit for your research. If you are not sure, read the appropriate sections before making your selection.

☒ Life sciences ☐ Behavioural & social sciences ☐ Ecological, evolutionary & environmental sciences

For a reference copy of the document with all sections, see nature.com/documents/nr-reporting-summary-flat.pdf

Life sciences study design

All studies must disclose on these points even when the disclosure is negative.

Sample size	N=5 subjects participated for ring electrode placement and configuration optimization analysis. N=10 subjects participated for blood pressure (BP) estimation analysis. Each participant went through a cold-pressor test, yielding in total of ~140 minutes (10,145 beat-to-beat data points) of continuous beat-to-beat BP and bioimpedance signal acquisition. N=3 subjects participated in PPG, bioimpedance comparison in skin-tone analysis. Each participant had 6 minute-long measurements, resulting in a total of 18 minutes of continuous PPG and bioimpedance signals.
Data exclusions	Not applicable.
Replication	For ring electrode optimization analysis, each setup was measured with minimum 3 iterations of 1 minute. For BP analysis, each participant's BP and bioimpedance data was measurement with multiple cold-pressor and recovery cycles (min. 2). For skin-tone analysis, each participant's data was measured for 3 iterations of 1 minute for each sensor placement.
Randomization	Not applicable.
Blinding	Not applicable.

Reporting for specific materials, systems and methods

We require information from authors about some types of materials, experimental systems and methods used in many studies. Here, indicate whether each material, system or method listed is relevant to your study. If you are not sure if a list item applies to your research, read the appropriate section before selecting a response.

Materials & experimental systems

n/a	Involved in the study	
☒	☐	Antibodies
☒	☐	Eukaryotic cell lines
☒	☐	Palaeontology and archaeology
☒	☐	Animals and other organisms
☒	☐	Clinical data
☒	☐	Dual use research of concern

Methods

n/a	Involved in the study	
☒	☐	ChIP-seq
☒	☐	Flow cytometry
☒	☐	MRI-based neuroimaging
